# Hereditary Talent

**Published:** 1857-08

**Authors:** 


*Hereditary Talent.* — The July number of the American Journal of Medical Sciences contains the Inaugural Thesis of Dr. Austin Flint, Jr., the son of the distinguished former editor of this journal. It was published by order of the faculty of the Jefferson Medical College, in which school Dr. Flint, Jr., graduated. Its subject is the "Phenomena of the Capillary Circulation," and exhibits the same devotion to *personal* research and inquiry, which characterize the father of the author. Dr. Flint, Jr., has commenced practice in this city, and we hope ere long to witness some confusion of names, which will render it difficult to tell whether the older or younger Flint is intended, when some good thing in medicine is noticed. However, from the facility with which the Junior whipped off an arm, the other morning, in our presence, we are inclined to think that his laurels may lie in the field of surgery; leaving to the Senior (not yet old enough to be "venerable,") his old province of practical medicine.

